# Mesenchymal Stem Cell Exosomes in Combination with Hydrogels for Osteoarthritis: From Exploratory Applications to Optimization and Translational Outlook

**DOI:** 10.3390/cimb48080764

**Published:** 2026-07-27

**Authors:** Xuemeng He, Menghang Guo, Huan Lian, Liang Chen

**Affiliations:** National Institutes for Food and Drug Control (NIFDC), No. 31, Huatuo Road, Daxing District, Beijing 102629, China; hxm20020605@163.com (X.H.); guomenghang@henu.edu.cn (M.G.); lianhuan@nifdc.org.cn (H.L.)

**Keywords:** osteoarthritis (OA), mesenchymal stem cell-derived exosomes (MSC-Exos), hydrogel, synergistic effect, clinical translation

## Abstract

Osteoarthritis (OA) is a chronic degenerative joint disorder characterized primarily by cartilage degradation and extensive inflammation. Its pathological progression is closely associated with the dysregulation of critical signaling pathways; consequently, the therapeutic targets of these pathways have become crucial for intervening underlying mechanisms of cartilage damage. Extensive laboratory studies have found that mesenchymal stem cell-derived exosomes (MSC-Exos) can deliver bioactive cargoes such as microRNAs (miRNAs) and antioxidant enzymes to modulate these targets, thereby exerting therapeutic effects that include mitigating inflammation, inhibiting chondrocyte apoptosis, maintaining extracellular matrix homeostasis, and promoting tissue repair. However, the clinical translation of MSC-Exos is significantly hampered by inherent limitations, including source variability, low stability, and rapid clearance from the joint cavity. Hydrogels, recognized for their excellent biocompatibility, three-dimensional biomimetic architecture, and controlled drug release capabilities, have been used for OA therapy. Therefore, this review summarizes current research on the combined application of exosomes and hydrogels for OA treatment, and proposes optimization strategies for exosome preconditioning, hydrogel material selection, and hydrogel functional design. These strategies aim to enhance exosome functionality as well as enable sustained responsive, and localized exosome release within the joint, thereby improving the therapeutic efficacy for OA.

## 1. Introduction

Osteoarthritis (OA) is a clinically prevalent chronic degenerative joint disease that results from a complex interplay of pathological mechanisms. Its core manifestations include the progressive destruction of articular cartilage and inflammatory infiltration of the synovium. Pathogenesis is closely associated with the dysregulation of key signaling pathways, including NF-κB, MAPK, mTOR, and Wnt/β-catenin, as well as the activation of endoplasmic reticulum stress and an imbalance in oxidative stress. These factors collectively drive a cross-tissue vicious cycle of pathological changes involving “bone resorption—inflammation—cartilage damage”, ultimately leading to irreversible joint dysfunction and severely impairing patients’ quality of life [1,2,3,4,5,6].

Current mainstream clinical treatments including nonsteroidal anti-inflammatory drugs, glucocorticoids, and joint replacement, primarily provide symptomatic relief and fail to halt the fundamental progression of cartilage degeneration or tissue damage. In recent years, biological therapies have attracted increasing attention as potential disease-modifying strategies for OA. Among them, platelet-rich plasma (PRP), which contains multiple growth factors and bioactive mediators, has been used clinically to relieve pain, modulate inflammation, and improve joint function in some patients with OA. However, its therapeutic efficacy remains variable, and its ability to reverse established cartilage degeneration is still limited [7]. Consequently, there is an urgent need for innovative therapeutic strategies that target pathological mechanisms, modulate the inflammatory microenvironment, and promote cartilage repair. Mesenchymal stem cell-derived exosomes (MSC-Exos) carry bioactive molecules such as microRNAs (miRNAs) and antioxidant enzymes. These exosomes exert potent anti-inflammatory effects, reduce chondrocyte apoptosis, maintain extracellular matrix homeostasis, and facilitate cartilage tissue repair, thereby demonstrating considerable therapeutic potential in OA treatment [8,9]. However, their short in vivo half-life, rapid clearance from the joint cavity and instability severely limit therapeutic efficacy and clinical translation [10].

Hydrogels, characterized by excellent biocompatibility, a three-dimensional biomimetic structure, tunable mechanical properties, and controlled drug release capabilities. They can not only simulate the extracellular matrix to improve the joint microenvironment but also serve as ideal carriers to achieve local sustained delivery and activity protection of exosomes [11]. Integrating exosomes with hydrogels to construct a combined system can not only address the bottleneck of in vivo exosome delivery but also exert the synergistic effects of anti-inflammation, maintenance of matrix homeostasis, and cartilage regeneration, thereby providing an innovative approach for precise and long-lasting OA treatment.

Therefore, this review provides an overview of the current research on exosome–hydrogel combination therapy for OA. Exosome candidates that have shown preliminary efficacy in targeting key pathogenic signaling pathways of OA are systematically compiled and analyzed in this paper, revealing that the rational design of an exosome–hydrogel-combined system hinges on the use of such functionally relevant exosomes. Subsequently, optimization strategies for exosome preconditioning, hydrogel material selection, and hydrogel functional design are delineated. These strategies aim to improve exosome functionality and achieve sustained, responsive, and localized exosome release within the joint, ultimately enhancing the therapeutic performance of the exosome–hydrogel combination product for OA treatment.

## 2. Materials and Methods

This review was conducted and reported in accordance with the PRISMA 2020 guidelines. The completed PRISMA 2020 checklist is provided as Supplementary Materials. The review protocol was not registered in a publicly accessible registry.

### 2.1. Literature Search

A systematic literature search was conducted in PubMed, Web of Science, Wanfang Data, and the China National Knowledge Infrastructure (CNKI) for studies published between January 2021 and July 2026. Medical Subject Headings (MeSH) and free-text terms were combined for PubMed in the title and abstract fields. The search strategies for each database were as follows:

PubMed: (“Exosomes”[TIAB] OR “Exosome”[TIAB]) AND (“Hydrogels”[TIAB] OR “Hydrogel”[TIAB]) AND (“Osteoarthritis”[TIAB] OR “OA”[TIAB]).

Web of Science: ((TI = (“Exosomes” OR “Exosome”)) OR AB = (“Exosomes” OR “Exosome”)) AND ((TI = (“Hydrogels” OR “Hydrogel”)) OR AB = (“Hydrogels” OR “Hydrogel”)) AND ((TI = (“Osteoarthritis” OR “OA”)) OR AB = (“Osteoarthritis” OR “OA”)).

Wanfang Data: (Title:(exosomes) OR Abstract:(exosomes)) AND (Title:(hydrogels) OR Abstract:(hydrogels)) AND ((Title:(osteoarthritis) OR Abstract:(osteoarthritis)) OR (Title:(OA) OR Abstract:(OA))).

CNKI: (TI = ‘exosome’ + ‘exosomes’ OR AB = ‘exosome’ + ‘exosomes’) AND (TI = ‘hydrogel’ + ‘hydrogels’ OR AB = ‘hydrogel’ + ‘hydrogels’) AND (TI = ‘osteoarthritis’ + ‘OA’ OR AB = ‘osteoarthritis’ + ‘OA’).

### 2.2. Inclusion and Exclusion Criteria

Published original research articles and reviews with complete data were considered eligible for inclusion. Abstract-only publications, conference papers, patents, duplicate publications, and records unrelated to the review topic were excluded.

Studies were excluded if they were outside the scope of OA treatment, unrelated to MSC-derived exosomes or hydrogel-mediated delivery, or focused primarily on veterinary application.

### 2.3. Screening and Data Extraction

Two investigators independently screened the retrieved records, first by title and abstract and subsequently through full text assessment. Any disagreement was resolved through discussion or consultation with a third reviewer.

**Figure 1:**
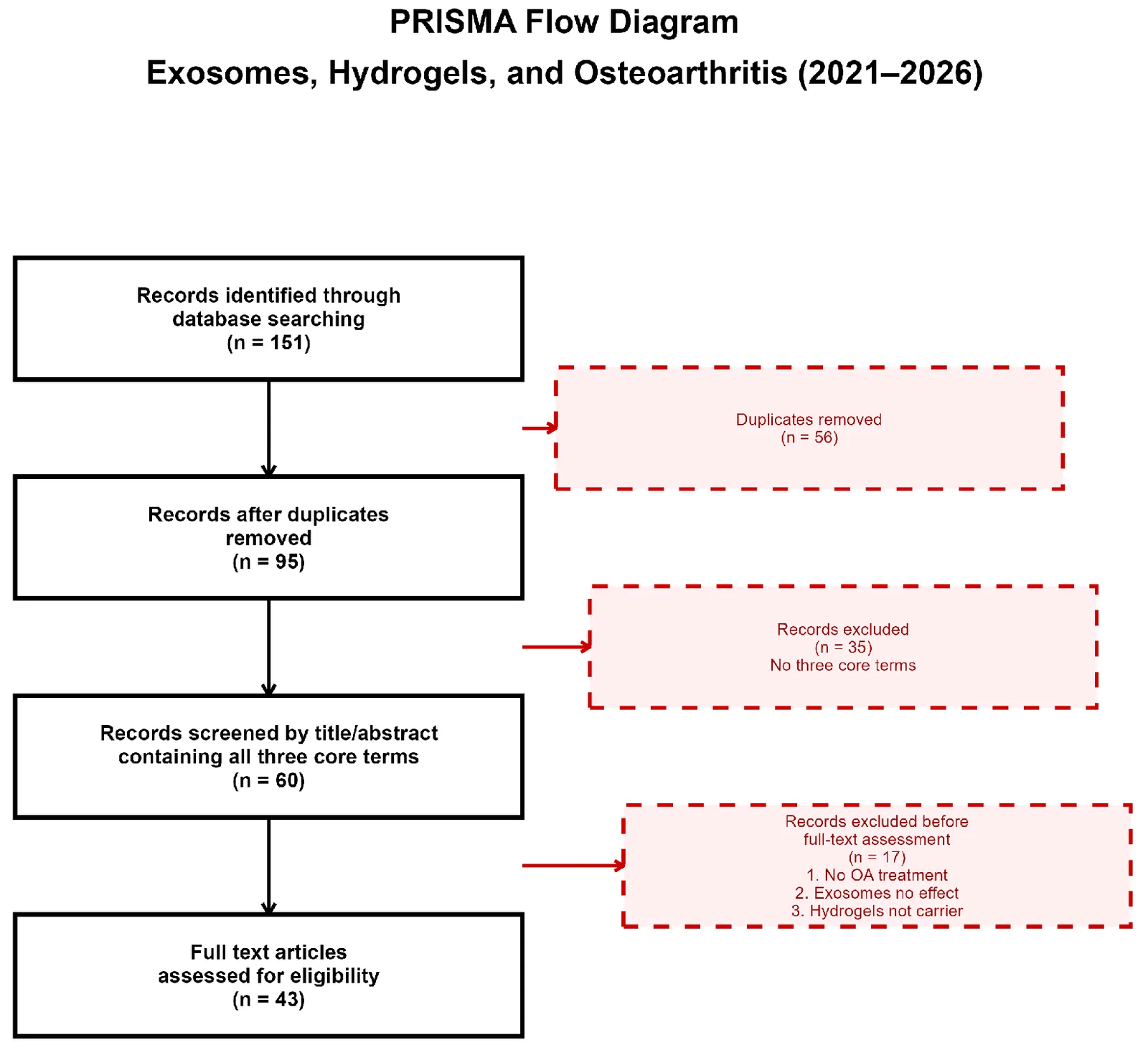
PRISMA flow diagram.

The initial search yielded 151 records, including 59 from PubMed, 54 from Web of Science, 18 from Wanfang Data, and 20 from CNKI. After duplicates were removed using reference management software, 95 unique records remained. Following title and abstract screening, 60 records that contained all three core terms were identified. A total of 43 records involving OA treatment, the therapeutic effects of exosomes, and the use of hydrogels as carriers proceeded to full text assessment. The detailed study selection process and results are presented in the PRISMA flow diagram (Figure 1).

## 3. Results

### 3.1. MSC-Exos Target Key Signaling Pathways in OA Pathogenesis

The onset and progression of OA are closely associated with the aberrant activation of multiple key cellular signaling pathways, among which the dysregulation of the NF-κB, MAPK, mTOR, and Wnt/β-catenin signaling pathways plays a core role [12,13]. NF-κB is a major inflammatory signaling pathway that promotes the production of pro-inflammatory cytokines and cartilage-degrading enzymes. MAPK signaling participates in cellular stress responses, inflammation, and chondrocyte catabolism. The mTOR pathway regulates cell metabolism, autophagy, and survival, while the Wnt/β-catenin pathway is involved in chondrocyte differentiation, ECM remodeling, and cartilage homeostasis.

**Figure 2:**
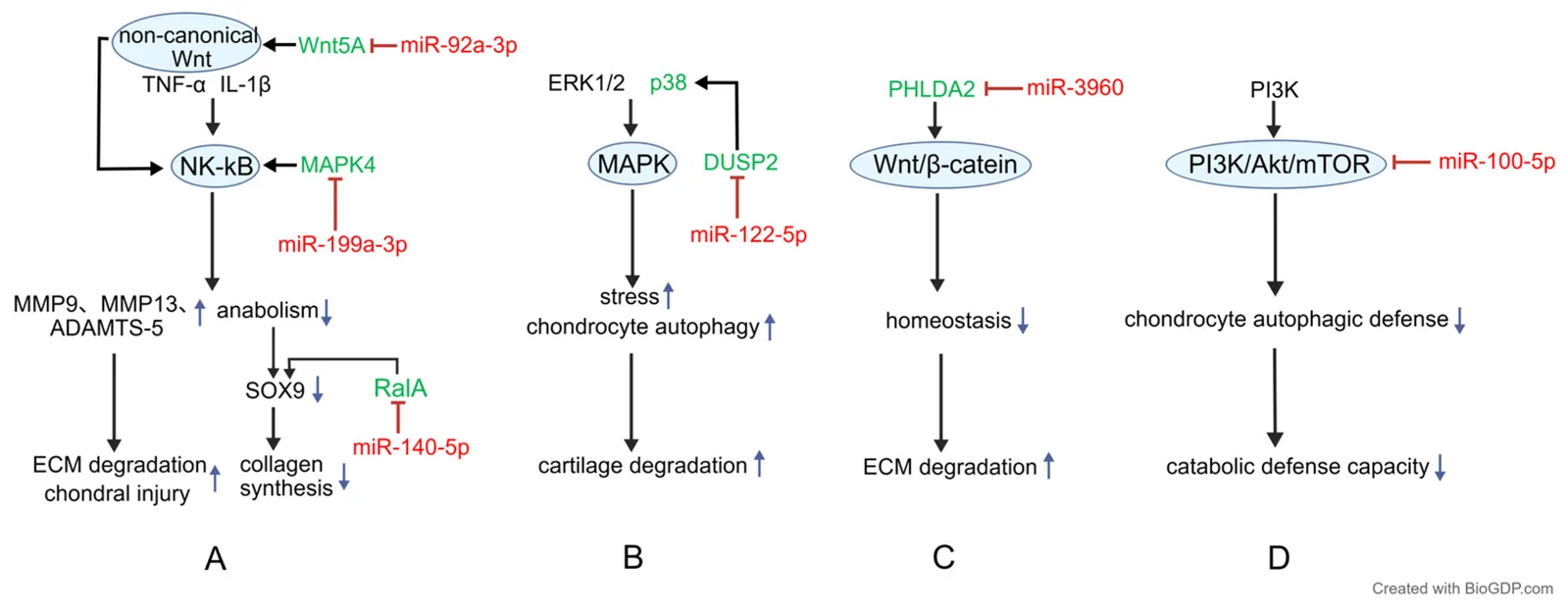
Key OA pathogenic pathways modulated by exosomes for therapy. Note: This figure summarizes four key signaling pathways, including the NF-κB (**A**), MAPK (**B**), Wnt/β-catenin (**C**), and mTOR (**D**) pathways, through which mesenchymal stem cell-derived exosomes (MSC-Exos) exert therapeutic effects in osteoarthritis (OA). Red short bar (┬): miRNA inhibits its target. Blue upward arrow (↑): Increased effect. Blue downward arrow (↓): Decreased effect. In this figure, miRNAs with functional roles are shown in red, and their target molecules or downstream effectors are shown in green. This figure was drawn by Xuemeng He using BioGDP [24] (Available at: https://www.BioGDP.com (accessed on 15 April 2026)).

MSC-derived exosomal miRNAs can regulate these pathways and thereby protect cartilage. miR-199a-3p from human umbilical cord MSC-derived exosomes directly targets MAPK4, a member of the MAPK family involved in inflammatory signal transduction. By inhibiting MAPK4, miR-199a-3p suppresses activation of the NF-κB pathway and reduces inflammatory responses in chondrocytes [14]. Similarly, miR-92a-3p from human bone marrow MSC-derived exosomes targets WNT5A, an upstream ligand of the non-canonical Wnt pathway. This regulation helps maintain cartilage development and homeostasis and inhibits WNT5A-mediated, NF-κB-dependent cartilage destruction [15]. In addition, miR-140-5p from synovial MSC-derived exosomes targets Ras-related protein RalA, a small GTPase involved in intracellular signaling. By suppressing RalA, miR-140-5p restores SOX9 expression, supports chondrocyte proliferation and migration, and maintains extracellular matrix (ECM) homeostasis [16]. as shown in Figure 2A. Regarding the MAPK pathway, exosomes derived from hypoxic-preconditioned human bone marrow mesenchymal stem cells (MSCs) can inhibit DUSP2 through miR-122-5p, abrogate its negative regulation on the ERK1/2 and p38 MAPK pathways, and then activate chondrocyte autophagy to promote OA cartilage repair [17], as shown in Figure 2B. In addition, miR-3960 in MSC-Exos exerts its effects by targeting and inhibiting PHLDA2. Given that PHLDA2 is positively correlated with SDC1 expression and Wnt/β-catenin pathway activation, this regulatory mode can further inhibit chondrocyte apoptosis and reduce ECM degradation [18], as shown in Figure 2C. Furthermore, miR-100-5p in exosomes derived from infrapatellar fat pad MSCs can bind to the 3′ untranslated region of mTOR, inhibit the mTOR signaling pathway, activate the chondrocyte autophagic defense system, and enhance the resistance of chondrocytes to catabolism [19], as shown in Figure 2D.

In addition, MSC-Exos can also exert regulatory effects on other pathological changes in the pathogenesis of OA. With respect to inhibition of chondrocyte apoptosis, miR-486-5p carried by adipose-derived mesenchymal stem cell exosomes (ADSC-Exos) suppresses the endoplasmic reticulum stress signaling pathway [20]. This is achieved through downregulation of GRP78, p-PERK, p-IRE1α, and CHOP expression, ultimately exerting protective and therapeutic effects on OA [20]. As for oxidative stress, functional antioxidant enzymes encapsulated in ADSC-Exos, including superoxide dismutase and catalase, directly scavenge excessive intracellular reactive oxygen species [21]. The resulting maintenance of redox homeostasis helps improve the chondrocyte microenvironment and retard OA progression [21]. With regard to maintenance of extracellular matrix homeostasis, MSC-Exos act primarily by transporting multiple miRNAs such as miR-140-5p and miR-92a-3p [22]. Specifically, they upregulate anabolic genes including collagen type II alpha 1 chain and aggrecan, while simultaneously downregulating catabolic genes such as matrix metalloproteinase 13 (MMP-13) and a disintegrin and metalloproteinase with thrombospondin motifs 5 (ADAMTS-5), thereby restoring the dynamic balance between synthesis and degradation [22]. In addition, MSC-Exos are also involved in the regulation of vascular responses. miR-181b in adipose-derived mesenchymal stem cell exosomes promotes local joint angiogenesis by upregulating hypoxia-inducible factor 1α (HIF-1α) and vascular endothelial growth factor (VEGF) [23], thus participating in the modulation of OA pathological progression [23].

### 3.2. Application of Hydrogels in OA Therapy

In the field of OA treatment, hydrogels not only act as versatile drug carriers that enable the long-term and controlled release of therapeutics but also exert multifunctional effects including lubrication, anti-inflammation, and cartilage repair.

#### 3.2.1. As a Drug Delivery System

Hydrogels facilitate sustained drug release following intra-articular injection for OA management. For instance, dexamethasone-loaded thermosensitive formaldehyde-glycerol-borax hydrogel (with a glycerol-to-chitosan volume ratio of 3:10) enables prolonged drug release [25]. Similarly, a cross-linked hyaluronic acid-dexamethasone hydrogel further reduces the cytotoxicity of the drug and synergistically exerts chondroprotective and anti-inflammatory effects [26]. Further, microfluidic and photopolymerization techniques have been used to fabricate injectable, cartilage-targeting gelatin methacrylate (GelMA) hydrogel microspheres, which are engineered to load reactive oxygen species-responsive drug nanoparticles. The system achieves long-term intra-articular retention, stimuli-responsive drug release, and enhances the survival and density of implanted ATDC5 mouse chondrogenic progenitor cells [27].

Beyond drug delivery, hydrogels can function as effective cell carriers. Studies have demonstrated that hyaluronic acid hydrogels enable targeted delivery of MSCs to the joint cavity while preserving cell viability and displaying outstanding biocompatibility [28]. Collectively, hydrogels have now been demonstrated to enable the delivery of small-molecule drugs, macromolecular biopeptides, and even cells into the joint cavity for OA treatment. Accordingly, hydrogels are promising carriers for the delivery of exosomes in OA therapy.

#### 3.2.2. Synergistic Therapeutic Effects Imparted by Hydrogel

Hydrogels exhibit diverse adjunctive therapeutic effects in the treatment of OA, attributed to their inherent material properties, including piezoelectricity, lubricity, chemical reactivity, and biocompatibility. For instance, an injectable, biodegradable piezoelectric hydrogel composed of short electrospun poly(L-lactic acid) nanofibers and a collagen matrix can autonomously generate localized electrical signals upon activation by ultrasound (40 kHz, 0.33 W/cm^2^). This electrical stimulation promotes chondrogenesis by inducing stem cells to secrete endogenous transforming growth factor-β1 (TGF-β1) [29]. Separately, hydrogels based on materials such as 2-methacryloyloxyethyl phosphorylcholine, chitosan, and gellan gum act as biomimetic lubricants. These hydrogels can alleviate joint inflammation or repair defective tissue by leveraging their inherent mechanical properties, such as compressive resistance and lubricity, thereby effectively reducing cartilage damage. Furthermore, Kim et al. designed a hybrid “click chemistry” hydrogel embedded with polymeric aggregates. This hydrogel is formed in situ via a click reaction between azide-functionalized hyaluronic acid and PLA-b-PEG-N_3_ polymeric aggregates, mediated by a nitric oxide (NO)-cleavable cross-linker. This hydrogel integrates both NO scavenging and sequential drug release capabilities, enabling synergistic treatment of rheumatoid arthritis through dual mechanisms [30]. On the other hand, an IEIK13 self-assembling peptide hydrogel, upon implantation, attracts the homing of bone marrow-derived mesenchymal stem cells (BM-MSCs), which subsequently differentiate into chondrocytes and secrete characteristic cartilage matrix, ultimately leading to OA alleviation [31]. When used as carriers for exosomes, these intrinsic material properties of hydrogels are poised to synergistically enhance the therapeutic efficacy of exosome-based OA therapy.

### 3.3. Exploratory Use of Hydrogels in Combination with MSC-Exos for OA Therapy

#### 3.3.1. Integration Approaches of MSC-Exos and Hydrogels

The integration of MSC-Exos with hydrogels is a pivotal strategy for achieving their synergistic therapeutic effect in OA. The methods of combination can be categorized into two main types: physical encapsulation [32,33,34,35,36,37,38,39,40,41,42,43] and chemical cross-linking [44,45,46,47,48].

Physical encapsulation involves directly mixing exosomes with a hydrogel matrix such as GelMA, functionalized hyaluronic acid, or composite hydrogels. Hydrogel formation is induced via visible/ultraviolet light crosslinking or Schiff base reactions to contain exosomes within the resulting three-dimensional network. For example, Pang et al. [39] mixed MSC-Exos with GelMA and a photoinitiator, then cross-linked the mixture using 405 nm UV light to form a GelMA-Exos composite hydrogel. Zhao et al. [42] physically mixed human Wharton’s jelly mesenchymal stem cell-derived small extracellular vesicles with solutions of alginate-aldehyde (ALG-CHO) and hydrazide-modified hyaluronic acid (HHA). The Schiff base reaction between the aldehyde groups in ALG-CHO and the amine groups in HHA enabled rapid in situ cross-linking of the mixture, forming a three-dimensional network that embedded the exosomes.

In contrast, chemical cross-linking method employs cross-linkers to form covalent bonds (e.g., disulfide bonds, amide bonds) between functional groups on the exosome surface. This covalent conjugation yields a more stable integration, which can significantly prolong the exosome release profile and minimize their diffusion loss from the joint cavity. For instance, researchers use allyl-L-glycine-modified CP05 peptide as a linker between the side chain of GelMA hydrogel and the CD63 protein on the exosome surface, anchoring the exosomes and ultimately forming a stable “GelMA-CP05-Exosome” composite system [48]. Cao et al. [44] integrated exosomes with a thiolated hyaluronic acid microgel by forming disulfide bonds between the thiol groups on the microgel and thiol groups on exosome surface proteins, alongside non-covalent interactions, creating a “two-phase” release system.

#### 3.3.2. Improvement of Joint Retention and Sustained Release of Exosomes by Hydrogel Carriers

The short in vivo half-life and poor intra-articular retention significantly limit their therapeutic efficacy. Hydrogel delivery systems can effectively address this problem by forming a stable drug reservoir within the joint, thereby prolonging exosome retention. Studies have confirmed that the exosome delivery system based on the mussel-inspired multi-responsive hydrogel, which uses chitosan and catechol-modified chitosan as the skeleton, combined with β-glycerophosphate disodium and dialdehyde-functionalized polyethylene glycol to form a thermosensitive, self-healing and adhesive multifunctional system. This system can form a stable long-term drug release depot in the articular cavity, significantly prolong the in vivo retention time of exosomes and enhance their stability, thereby fully exerting the long-term therapeutic effect of exosomes [49]. Research has also demonstrated that a thermosensitive injectable hydrogel constructed via in situ cross-linking of Pluronic F-127 and hyaluronic acid can serve as a sustained-release carrier to persistently retain adipose-derived MSC-Exos at the site of cartilage injury, effectively amplifying their reparative effects [50]. For instance, Yang et al. [51] fabricated hyaluronic acid methacrylate/gelatin methacrylate (HAMA/GelMA) hydrogel microcarriers incorporated with stem cell recruitment peptide SKPPGTSS and exosomes, which enable gentle exosome encapsulation and sustained release for over 8 days. The delivery system developed by Zhou et al. [34], based on a composite hydrogel of GelMA and oxidized chondroitin sulfate loaded with BMSC-Exos, achieved sustained exosome release for 14 days with a cumulative release exceeding 80%. Another study has shown that thermosensitive hydrogels constructed with an optimal ratio of Poloxamer 407/188 as a carrier, loaded with platelet-derived exosomes, enable sustained release of exosomes for up to 28 days. In vivo experiments further confirmed that this composite increases the local retention of exosomes and slows subtalar arthritis progression by inhibiting chondrocyte apoptosis and hypertrophy while promoting chondrocyte proliferation and potential stem cell recruitment [52].

### 3.4. Optimization of Hydrogels in Combination with MSC-Exos for Enhanced Therapeutic Efficacy

#### 3.4.1. Optimization Through Exosome Preconditioning

Preconditioning techniques are employed to optimize the bioactive cargo of MSC-Exos for OA therapy. These techniques primarily fall into two categories-biostimulation and culture environment modulation-which trigger the reprogramming of MSC transcriptomes, proteomes, and metabolomes.

As shown in Table 1, biostimulation-based preconditioning centers on cytokine stimulation. TGF-β1 stimulation significantly upregulates miR-135b expression in exosomes secreted by neonatal rat bone marrow MSCs. This miR-135b promotes the polarization of synovial macrophages towards the M2 phenotype by targeting mitogen-activated protein kinase 6 (MAPK6), thereby ameliorating cartilage damage. Animal studies have confirmed that these preconditioned exosomes can significantly reduce inflammation and cartilage damage in OA models [53]. Similarly, TNF-α preconditioning promotes exosome secretion by activating the PI3K/AKT signaling pathway and upregulating ATG16L1 expression. These TNF-α-preconditioned exosomes are enriched with low-density lipoprotein receptor-related protein 1 (LRP1) and can degrade MMPs and ADAMTSs in chondrocytes to protect the ECM. In vivo studies have shown that they alleviate pathological changes and restore gait abnormalities in OA mice more effectively than non-preconditioned exosomes [54]. Furthermore, parathyroid hormone (1-34) preconditioning of bone marrow MSCs yields exosomes with high expression of let-7a-5p, which researchers have identified as the core functional miRNA. This let-7a-5p targets and inhibits IL-6 expression, blocking the IL-6/STAT3 signaling pathway, and subsequently significantly promotes chondrocyte proliferation and migration while reducing ECM degradation (inhibiting enzymes like MMP-9 and MMP-13, and elevating COL-2 and AGG expression) [55].Culture environment modulation-based preconditioning relies on oxygen tension regulation and matrix microenvironment modification to achieve its effects. The secretome from MSCs preconditioned under hypoxia (1% or 5% O_2_), compared to normoxia (20% O_2_), significantly enhances chondrocyte proliferation, migration, and the deposition of sGAG and type II collagen in vitro. It also suppresses IL-1β-induced chondrocyte senescence, the expression of inflammatory factors (IL-1β, IL-6), and ADAMTS5 activity, while downregulating the transcription and secretion of M1 macrophage-related inflammatory factors, with 1% O_2_ emerging as the optimal condition [56]. Exosomes derived from decellularized extracellular matrix (dECM)-preconditioned BMSCs, where dECM is a biomimetic culture scaffold that removes cells and retains only natural matrix, more potently promote IL-1β-induced chondrocyte proliferation, anabolism, and migration while inhibiting apoptosis. In a destabilization of the medial meniscus mouse model, these exosomes enhance cartilage regeneration and delay OA progression by delivering upregulated miR-3473b, which targets phosphatase and tensin homolog deleted on chromosome ten (PTEN) to activate the PTEN/AKT signaling pathway [57]. Additionally, exosomes (3D-Exos) produced by MSCs cultured in a three-dimensional (3D) GelMA hydrogel system exhibit superior anti-inflammatory, pro-proliferative, and tissue-remodeling properties compared to those from traditional two-dimensional (2D) culture [58].

#### 3.4.2. Functional Optimization of Hydrogels

To further enhance the intra-articular retention, targeting ability, and therapeutic efficacy of MSC-Exos in combination with hydrogel in OA intervention, it is expected to apply the latest advances in hydrogel-based delivery system research. Specifically, as shown in Table 2, by screening hydrogel materials and optimizing hydrogel release functions, more controlled sustained release, barrier-penetrating delivery, and stimuli-responsive release of exosomes can be achieved, together with exploiting the cartilage-repair promoting properties of hydrogel materials, to collectively enhance the therapeutic efficacy of hydrogel combined with exosomes for OA treatment.

Extensive research has confirmed that rationally engineered hydrogel carriers can remarkably prolong the release duration of exosomes. Through the selection of different hydrogel materials and the incorporation of exosomes into these hydrogels, the sustained intra-articular release duration of exosomes can be prolonged from 8 days to 28 days [32,49,50].

To achieve effective delivery to the diseased subchondral bone, design optimizations must address the dense structure and highly negatively charged matrix of cartilage. Hydrogel can be carried on the surface threads of the Stimulation Needle (ST-needle), which is featured with a threaded tip structure [59]. The hydrogel is protected by the threads as the cartilage barrier is penetrated by the needle, after which the hydrogel is swollen and drugs are released upon contact with bodily fluids. Experimental results showed that this system successfully delivered drugs to the subchondral bone in OA rats, with efficacy significantly superior to the intra-articular injection control group. This minimally invasive and precise delivery strategy can be fully applied to the hydrogel combined with exosomes.

Stimuli-responsive on-demand release designs leverage the intrinsic responsiveness of hydrogels to achieve precise, demand-driven release. Modulating polymer composition or incorporating stimuli-responsive moieties (e.g., pH-sensitive acrylates [60] or temperature-sensitive PLGA-PEG-PLGA triblock copolymers [61]) enables researchers to customize release profiles [62].Delivery systems based on mechano-active biomaterials can sensitively respond to mechanical signals, whereas wireless on-demand delivery systems triggered by exogenous stimuli (e.g., acoustic waves, electric fields, magnetic fields, or electromagnetic radiation) are attracting increasing attention. Du et al. developed a multifunctional conductive hydrogel composed of chitosan, polyvinyl alcohol, and graphene oxide, with which the release rate could be regulated by applying a 0–2 V voltage through a wireless circuit [63], offering a new paradigm for OA therapy.

Furthermore, the mechanical properties of hydrogels like polyethylene glycol (PEG)-based hydrogels [64] and PVA/PEG composite hydrogels [65] can be precisely tuned by adjusting precursor parameters or constructing biomimetic structures. Hydrogels with specific mechanical properties can meet the mechanical requirements for cartilage repair. The diverse intrinsic properties of hydrogel materials (such as piezoelectricity, lubricity, chemical reactivity, etc.) can play an auxiliary role, thereby being exploited to enhance the therapeutic efficacy of the combined hydrogel–exosome system for OA treatment.

## 4. Discussion

The combination of exosomes and hydrogels represents an innovative approach for OA therapy, but several significant challenges remain to be addressed to advance its clinical translation.

First, the tissue source of MSC-Exos is an important factor affecting their therapeutic potential. Bone marrow-derived MSC-Exos have been widely studied and show strong capacity to regulate cartilage homeostasis and inflammatory responses, but bone marrow collection is relatively invasive and donor-dependent. Adipose-derived MSC-Exos are easier to obtain, have abundant tissue sources, and display notable anti-inflammatory and antioxidant properties. Umbilical cord-derived MSC-Exos can be obtained non-invasively and have advantages in proliferative capacity, immunomodulatory activity, and standardized large-scale production. However, to truly achieve source selection based on OA pathological characteristics, a deeper understanding of the molecular pathological heterogeneity of OA is first required. OA is increasingly recognized as comprising at least three pathological subtypes with distinct molecular characteristics: the bone remodeling subtype (C1) enriched with pathways related to skeletal muscle organ development; the immunemetbolic subtype (C2) focused on metabolic processes and immune response pathways, with significantly higher infiltration of M0 macrophages and activated mast cells compared to other subtypes; and the cartilage degradation subtype (C3) closely associated with pyroptosis and cell death processes [66]. Therefore, after identifying the specific therapeutic targets at the molecular pathology level for different subtypes of OA, appropriate exosomes should be selected, or engineered to contain cargoes (e.g., miRNAs) that can modulate these targets. Accordingly, MSC-Exos from different sources, owing to their intrinsic functional differences, may be suitable for distinct OA subtypes: adipose-derived MSC-Exos, with their prominent anti-inflammatory and antioxidant properties, may be more appropriate for oxidative stress-dominant pathological conditions; umbilical cord-derived MSC-Exos, with their outstanding immunomodulatory activity and advantages in standardized production, may be more suitable for immune-metabolic OA; and bone marrow-derived MSC-Exos, with their relatively strong capacity in regulating cartilage homeostasis, may be more appropriate for cartilage degradation-dominant OA.

Moreover, even when source selection is optimized based on OA subtypes, conventional animal models inadequately recapitulate the complex pathophysiology of human OA, which severely impedes objective efficacy assessment of exosome–hydrogel combinations and also hampers reliable extrapolation to clinical settings. To date, the vast majority of therapeutic efficacy data for MSC-Exos in OA have been derived from surgically or chemically induced rodent models. Notably, traumatic injury accounts for merely approximately 12% of human OA cases in clinical contexts; however, these rodent models are typified by acute, rapidly progressive lesions driven by overt mechanical or chemical insults, which fundamentally diverge from the insidious, multifactorial, spontaneous degenerative trajectory characteristic of human OA. Such discrepancies frequently engender systematic overestimation of in vivo therapeutic potency in rodent systems. Although large animal models more closely approximate human joint anatomy and biomechanics, their prohibitive husbandry expenses and protracted breeding cycles preclude widespread implementation. Moreover, the short-term histological endpoints conventionally adopted in animal investigations remain inadequate to satisfy the stringent requirements for long-term safety profiling and functional outcome assessment mandated in human clinical trials [67].

Second, the potential side effects caused by non-therapeutic components in exosomes need to be controlled and minimized. In addition to miRNAs that act on OA targets, exosomes contain a complex mixture of other components, including non-targeting miRNAs and other biomolecules. The composition and potential of these non-therapeutic contents require further characterization. For instance, mesenchymal stem cell-derived exosomes (MSC-Exos) can be recognized by immune cells such as macrophages, dendritic cells (DCs), T cells, and B cells [68]. MSC-Exos can induce over-activation and enhance the immunosuppressive function of myeloid-derived suppressor cells by activating the JAK2/STAT3 and TLR4 signaling pathways, potentially weakening the body’s immune surveillance and defense capabilities [69]. Furthermore, MSC-Exos selectively encapsulate various miRNAs (e.g., miR-223-3p, miR-142-3p, miR-21-5p, miR-126-3p). Upon delivery to DCs, these miRNAs target and degrade markers of maturation (CD83), cytokines (IL-6, IL-12A), and signaling pathway-related genes (Tsc1, CCR7), while also inhibiting CD38 expression. This triggers a cascade that ultimately hinders DC maturation, resulting in a “semi-mature” DC phenotype [70]. MSC-Exos can be internalized by activated CD19^+^/CD86^+^ B cells [71] and, via miR-155-5p, downregulate the PI3K/Akt signaling pathway [72]. This suppresses B cell proliferation, differentiation, antibody production, and memory B cell maturation, thereby reducing B cell activation capacity. MSC-Exos can also act on adenosine signaling transduction; adenosine possesses potent immunosuppressive effects, which can inhibit T cell proliferation in vitro [73]. The inhibitory effects on immune cells vary among exosomes from different sources, and excessive suppression may disrupt immune homeostasis and exacerbate OA. Moreover, the effects of MSC-Exos are strongly influenced by the local immune microenvironment. Although MSC-Exos are generally regarded as immunomodulatory, inappropriate or excessive immune regulation may theoretically disturb immune homeostasis. This may increase the risk of allergic reactions, autoimmune responses, or impaired host defense during infectious diseases. Therefore, future translational studies should not only evaluate cartilage repair and anti-inflammatory efficacy, but also systematically assess immunogenicity, immune safety, infection-related risks, biodistribution, dose–response relationships, and long-term outcomes.

Third, achieving precise control over exosome release from hydrogels is a critical hurdle for their combined application. The release kinetics need to be highly synchronized with the pathological progression of OA. Excessively rapid release leads to swift clearance of exosomes and transient efficacy, while overly slow release may fail to achieve therapeutic concentrations. Physical encapsulation of exosomes within hydrogels is prone to exosome leakage [74], whereas chemical conjugation risks damaging the exosomal membrane and internal bioactive molecules. The in vivo stability and response sensitivity of existing “smart” hydrogels (e.g., temperature-responsive or enzyme-responsive) lack sufficient validation in clinical trials [75].

Despite these delivery challenges, from a translational medicine perspective, injectable, biodegradable, biocompatible, and mechanically adaptable hydrogels may hold the greatest promise for OA therapy. Natural hydrogels, including hyaluronic acid, collagen, gelatin, chitosan, and alginate, can mimic certain features of the cartilage extracellular matrix and show good biocompatibility. Among them, hyaluronic acid-based hydrogels are particularly attractive because hyaluronic acid is already widely used in intra-articular therapy and possesses inherent lubrication and chondroprotective properties. When combined with MSC-Exos, hyaluronic acid-based hydrogels may not only serve as a viscosupplement but also as a sustained delivery platform that prolongs exosome retention in the joint cavity and potentially synergizes with the immunomodulatory and regenerative functions of exosomes. Gelatin methacryloyl and collagen-based hydrogels provide extra-cellular matrix-like microenvironments and tunable mechanical support, while thermosensitive hydrogels allow minimally invasive injection and in situ gel formation. The established clinical safety profiles and regulatory pathways of these biomaterials, particularly hyaluronic acid derivatives, may substantially lower the translational barriers for exosome–hydrogel combination products. Collectively, these characteristics make them suitable carriers for improving the local retention and sustained release of MSC-Exos in the joint cavity, representing a practical and clinically accessible strategy for OA therapy.

Beyond the aforementioned challenges at the biological and material levels, multiple bottlenecks—including industrial-scale manufacturing, storage stability, terminal sterilization, quality control, regulatory approval, and commercial translation—collectively impede the clinical adoption of this therapeutic strategy. To date, only a limited number of registered clinical trials have evaluated injectable exosome–hydrogel hybrid formulations for OA, and none of the candidate products have advanced to Phase III trials [76,77]. Safety risks pervade every stage of exosome fabrication and constitute a core barrier to clinical translation. Microbial contamination (e.g., mycoplasma and viruses) during cell culture severely compromises exosome quality and biosafety [78]. Residual reagents such as iodixanol [79] and polyethylene glycol [80] from the separation and purification process, if not thoroughly eliminated, can trigger allergic responses or immunotoxic effects [81,82]. Moreover, exosomes intrinsically carry major histocompatibility complex (MHC) molecules capable of eliciting immune responses; physical or chemical manipulations during manufacturing may further alter surface protein conformations, thereby exacerbating immunogenicity [83].

With respect to large-scale production, conventional two-dimensional (2D) culture systems and open manual workflows suffer from pronounced batch-to-batch variability, prohibitive costs, and unstable yields, substantially hindering industrial scale up. Although transitioning from 2D static culture to bioreactor platforms can improve exosome output, such systems are plagued by increased technical complexity and excessive resource consumption [84]. Regarding storage, repeated freeze–thaw cycles may rupture exosomal membranes and trigger leakage of intraluminal cargo. Lyophilization enables ambient-temperature preservation but its detrimental effects on exosome bioactivity remain to be systematically characterized [85]. Conventional sterilization methods often compromise the structural integrity of extracellular vesicles (EVs), and low-damage protocols specifically tailored for EV-laden hydrogels are currently unavailable.

In addition, unified quality control specifications are lacking for EV-hydrogel composite systems, and universally accepted acceptance criteria have not been established for critical quality attributes, including vesicle particle size, surface biomarkers, and residual impurities generated during fabrication. Notably, standardized characterization pipelines for EV quantification remain absent; routine assessments rely merely on basic detection of CD9/CD63 surface markers and particle size distribution, with no consensus on potency quantification or impurity limit control, leading to substantial inter-batch heterogeneity. From regulatory and commercial perspectives, the jurisdictional oversight of such biomaterial composite products remains ambiguous. As combined biological-device combination products, EV-hydrogel (EVH) constructs are classified inconsistently across global regulatory authorities. Meanwhile, exorbitant costs arising from large-scale manufacturing and cold-chain logistics, coupled with pricing challenges, further obstruct real-world clinical implementation [86].

In summary, biosafety concerns and multi-faceted translational barriers markedly delay the commercialization and widespread clinical adoption of exosome–hydrogel therapeutic platforms. Future research should advance in concert across multiple dimensions—including mechanistic understanding, material design, safety evaluation, and manufacturing processes—to translate this promising strategy into tangible clinical benefits for OA patients.

## 5. Conclusions

The dysregulation of interconnected signaling pathways constitutes the core molecular basis for OA progression. Exosomes can precisely target pathogenic molecular targets by delivering bioactive molecules such as miRNAs and antioxidant enzymes. Meanwhile, hydrogels not only serve as a delivery system to achieve sustained exosome release and local retention, but also provide adjuvant effects in promoting cartilage repair. However, the standalone application of exosomes faces limitations such as short in vivo half-life, rapid clearance from the joint cavity, and insufficient stability, whereas hydrogels alone lack active targeting and bioactivity regulatory capacity. Therefore, their combination is expected to yield superior therapeutic outcomes, forming a synergistic effect in anti-inflammation, maintenance of matrix homeostasis, inhibition of chondrocyte apoptosis, and promotion of cartilage regeneration, thereby providing a promising direction for breaking through the bottleneck of OA treatment. Furthermore, through optimization strategies such as exosome preconditioning and functional design of hydrogels, the synergistic therapeutic efficacy of the combined system can be significantly enhanced.

However, to achieve effective OA treatment, it is essential to first identify therapeutic targets specific to distinct molecular pathological subtypes of OA. Subsequently, by selecting MSC sources, MSC-Exos loaded with miRNAs that act on these targets can be obtained, or cell engineering strategies can be employed to enable MSC-Exos to load and even enhance the loading of such miRNAs. Meanwhile, potential side effects caused by non-therapeutic components within exosomes must be controlled and minimized. On the other hand, the selection and engineering design of hydrogels should also be optimized to achieve the desired release profile. Only in this way can the challenges in clinical translation of this combined system be overcome.

## Figures and Tables

**Table 1:** Summary of exosome preconditioning strategies.

Optimization Strategies	Examples	Principal Therapeutic Outcomes	Advantages	Animal Model
Biological Stimulation	TGF-β1	Upregulation of exosomal miR-135b targets MAPK6.	Intact exosome membrane, unimpaired bioactivity	Modified Hulth SD rat OA model
	TNF-α	Exosomes enriched in LRP1 activate the PI3K/AKT pathway.	Mild preconditioning conditions	DMM C57BL/6 mouse model
	PTH (1-34)	High expression of exosomal let-7a-5p inhibits the IL-6/STAT3 pathway.	circumvent the disadvantages of stem cell therapy	Collagenase II-induced rat OA model
Modulation of MSC Culture	Hypoxic pretreatment (1% O_2_)	Promotes chondrocyte proliferation and inhibits inflammation.	simple to implement with low cost	critical-sized rat osteochondral defect model
	Remove cellular components while preserving the native extracellular matrix. (dECM)	Increased expression of miR-347b suppresses PTEN, thereby activating the PTEN/AKT signaling pathway.	Mature, batch-processabl	DMM C57BL/6 mouse model
	3D culture	The expression of miR-150-5p was increased, which targeted PDCD4 and suppressed the release of pro-inflammatory cytokines.	Significant improvement in exosome yield	Composite rabbit model with corneal stromal defect combined with partial limbal stem cell deficiency (LSCD)

**Table 2:** Summary of hydrogel optimization strategies.

Examples	Principal Therapeutic Outcomes	Advantages	Animal Model	Exosome Loading Method	Release Duration
HAMA/GelMA hydrogel,	Enhanced Sustained Release	Simple and rapid fabrication, low-temperature system without organic solvents	Surgically induced traumatic knee OA model in SD rats	Premixing in situ encapsulation	~60% released within 8 d, monitored for 0–16 d
GMOCS hydrogel	Enhanced Sustained Release	Biomimetic cartilage matrix mechanically matched to joint loads	medial collateral ligament and medial meniscus transection-induced OA rat model	Physical coblending and in situ encapsulation	In vitro sustained release of exosomes over 14 days
ST-needle combined with hydrogels	Barrier-Penetrating Targeted Delivery	Superior puncture penetrability to break through the cartilage physical barrier	induce osteoarthritis in rats, rabbits and pigs	_	_
Responsive release with pH/temperature-sensitive moieties; CS/PVA/GO composite conductive hydrogel (voltage-controlled drug release)	Stimulus-Responsive On-Demand Release	_	_	_	_
PEG-based, PVA/PEG composite hydrogels	Mechanical Property Regulation	Tunable mechanical properties over a wide range	_	_	_

## Data Availability

No new data were created or analyzed in this study. Data sharing is not applicable to this article.

## Supplementary Materials

The following supporting information can be downloaded at: https://www.mdpi.com/article/10.3390/cimb48080764/s1.
